# Cost analysis of hospital infections in a training hospital

**DOI:** 10.1186/1753-6561-5-S6-P248

**Published:** 2011-06-29

**Authors:** G Ozbek, BB Özkaptan, IY Avcı, C Açıkel, L Gorenek, CP Eyigün

**Affiliations:** 1 Department of Infection Diseases and Clinical Microbiology, Gulhane Military Hospital, Ankara, Turkey; 2 School of Nursing, Sinop University, Sinop; 3 Department of Public Health, Gulhane Military Hospital, Ankara, Turkey

## Introduction / objectives

In our study; we investigated the effect of the hospital acquired infections in Gulhane Military Medical Faculty Training Hospital, in Turkey, on hospitalization period, mortality, and additional cost caused by these infections. Additionally; we tried to define the hospital infections types having the highest cost.

## Methods

Ninety patients with hospital acquired infection were included in the study. Ninety patients without hospital acquired infection one to one matched and having similar characteristics with study group were chosen as control. All expenditures for consumables were assessed in cost analysis.

## Results

In our study; it was detected that, the hospital acquired infections developed averagely on 18^th^ day of hospitalization, and that, the hospitalization duration increased 16.1 days due to these infections and the mortality was 14.5% higher. The additional cost per patient was approximately calculated 4435$. It was detected that, patients with hospital acquired infection caused 83.4$ of additional expenditure daily, and for these patients the highest cost were for consumables and drugs used. It was calculated that, 84% of increase in the drug cost was caused by additional antibiotics used. The cost increase in ventilation association pneumonia, in blood stream infection and in urinary system infection were more than that of other types of hospital acquired infections.

## Conclusion

There have been several studies in developed countries, unfortunately in our country there is limited information about the cost analysis of the hospital acquired infections. This significance of this study is its being the first in Turkey due to its inclusion and context.

## Disclosure of interest

None declared.

